# Association between the neutrophil-to-lymphocyte ratio and preeclampsia: a systematic review and meta-analysis of observational studies

**DOI:** 10.3389/fpubh.2026.1865278

**Published:** 2026-06-30

**Authors:** Xinqian Li, Duqin Chen, Ping Che, Yangming Liu

**Affiliations:** 1 Bioscience, School of Health Industry, Chongqing Vocational College of Applied Technology, Chongqing, China; 2 Department of Obstetrics and Gynecology, The Second People’s Hospital of Yubei District, Chongqing, China; 3 Department of Hepatobiliary Surgery, Shapingba Hospital affiliated to Chongqing University (Shapingba District People’s Hospital of Chongqing), Chongqing, China; 4 Department of Obstetrics, The Affiliated Banan Hospital of Chongqing Medical University (People’s Hospital of Banan District), Chongqing, China

**Keywords:** inflammation, meta-analysis, neutrophil-to-lymphocyte ratio, observational studies, preeclampsia, pregnancy, systematic review

## Abstract

**Background:**

Preeclampsia is a pregnancy-specific hypertensive disorder associated with substantial maternal and perinatal morbidity. The neutrophil-to-lymphocyte ratio (NLR) has emerged as a potential inflammatory biomarker, but existing evidence remains inconsistent.

**Methods:**

PubMed, Embase, and Web of Science Core Collection were searched from database inception to 9 March 2026. Observational studies comparing NLR between women with preeclampsia and normotensive pregnant controls were included. Study quality was assessed using the Newcastle-Ottawa Scale. Random-effects meta-analysis was used to calculate pooled standardized mean differences (SMDs), with Hartung-Knapp adjustment applied as a sensitivity analysis. Publication bias was assessed using funnel plot inspection, Egger’s test, and Begg’s test.

**Results:**

Fourteen studies were included in the qualitative synthesis and eleven in the quantitative synthesis. The pooled analysis demonstrated significantly higher NLR levels in women with preeclampsia (SMD = 0.76, 95% CI 0.54–0.98; *I*^2^ = 84.8%). Subgroup analysis showed significant associations both in studies measuring NLR in the first trimester or early pregnancy (SMD = 0.81, 95% CI 0.48–1.14; *I*^2^ = 88.7%) and in studies measuring NLR at other time points (SMD = 0.73, 95% CI 0.40–1.06; *I*^2^ = 83.4%). The test for subgroup differences was not statistically significant (*p* = 0.743), although this comparison was limited by the small number of studies in each subgroup. Formal tests did not detect statistically significant publication bias, but their power was limited by the small number of studies.

**Conclusion:**

Maternal NLR was significantly higher in women with preeclampsia than in normotensive pregnant controls. However, because the included studies were observational and showed substantial heterogeneity, and because diagnostic accuracy metrics and clinically actionable thresholds were not synthesized, NLR should not be interpreted as a stand-alone diagnostic or predictive marker. Further prospective studies with standardized sampling windows, prespecified cut-off values, and adjustment for key confounders are needed.

**Systematic review registration:**

Identifier PROSPERO CRD420261333930.

## 1 Introduction

Preeclampsia affects approximately 2–8% of pregnancies worldwide and remains a major cause of maternal and perinatal morbidity and mortality (1–4). According to current clinical guidelines, it is characterized by new-onset hypertension after 20 weeks of gestation accompanied by proteinuria and/or maternal organ dysfunction (5). Increasing evidence suggests that exaggerated maternal inflammatory responses contribute to the pathogenesis of the disease (6–11).

The neutrophil-to-lymphocyte ratio (NLR) reflects the balance between innate inflammatory activation and adaptive immune regulation and has been widely investigated as a marker of systemic inflammation (12–14). Several primary studies have explored the relationship between NLR and preeclampsia; however, the reported findings have been inconsistent (15–28).

Therefore, this study aimed to systematically review the available literature and quantitatively synthesize the evidence regarding the association between NLR and preeclampsia.

## 2 Methods

### 2.1 Study design and registration

This systematic review and meta-analysis was conducted in accordance with the PRISMA 2020 statement (29) and relevant methodological guidance from the Cochrane Handbook (30). The completed PRISMA 2020 checklist is provided as Supplementary File 1. The review was registered in the International Prospective Register of Systematic Reviews (PROSPERO; CRD420261333930). The PROSPERO record was created on 6 March 2026 and last edited on 28 May 2026. Preliminary literature screening and data organization had been initiated before registration; however, the final database search was completed on 9 March 2026. Therefore, at the time of PROSPERO registration, the final eligible study set, verified extraction dataset, and quantitative synthesis had not yet been finalized. No study had completed finalized, verified data extraction for quantitative synthesis before PROSPERO registration (0/14 studies, 0%).

### 2.2 Search strategy

PubMed, Embase, and Web of Science Core Collection were systematically searched from database inception to 9 March 2026. The search strategy combined terms related to preeclampsia and NLR, including “preeclampsia,” “pre-eclampsia,” “pregnancy-induced hypertension,” “neutrophil-to-lymphocyte ratio,” “neutrophil lymphocyte ratio”, and “NLR.” Reference lists of relevant articles and reviews were also checked to identify additional eligible studies.

### 2.3 Eligibility criteria

Studies were considered eligible if they met the following criteria: (1) observational study design; (2) inclusion of women with preeclampsia and normotensive pregnant controls; (3) reporting of NLR values or sufficient data to calculate effect estimates; and (4) human studies published in English. Reviews, editorials, conference abstracts without sufficient data, case reports, animal studies, duplicate publications, and studies without extractable NLR data were excluded from quantitative synthesis. Studies lacking continuous NLR data but otherwise relevant to the topic were retained for qualitative synthesis when appropriate.

### 2.4 Study selection and data extraction

Two reviewers independently screened titles, abstracts, and full texts. The extraction form was discussed and standardized before formal extraction. Disagreements were resolved through discussion or consultation with a third reviewer. Formal inter-reviewer agreement statistics were not calculated. Extracted information included first author, publication year, country, study design, sample size, participant groups, timing of NLR measurement, preeclampsia definition and severity classification when available, adjusted confounders when reported, key exclusion criteria when available, and NLR summary statistics. For studies reporting multiple relevant groups or time points, the most clinically appropriate comparison between preeclampsia cases and normotensive pregnant controls was extracted.

### 2.5 Quality assessment

The methodological quality of included studies was assessed using the Newcastle-Ottawa Scale (NOS), which evaluates participant selection, comparability of study groups, and exposure or outcome ascertainment. Studies scoring 7–9 points were categorized as high quality, those scoring 5–6 points as moderate quality, and those scoring <5 points as low quality. Domain-level NOS scoring and the main risk-of-bias concerns for each study are presented in Supplementary Table S4.

### 2.6 Statistical analysis

Standardized mean differences (SMDs) with 95% confidence intervals (CIs) were used as the effect measure because NLR values were reported on a continuous scale across studies. Random-effects meta-analysis was performed using the DerSimonian-Laird method as the primary model (31). Hartung-Knapp adjustment was applied as a sensitivity analysis to account for uncertainty in between-study variance estimation. Because heterogeneity was substantial and the number of studies was limited, additional sensitivity analyses were performed using restricted maximum likelihood (REML) and Paule-Mandel estimators for between-study variance. Heterogeneity was assessed using the *I*^2^ statistic and τ^2^ (32). A fixed-effect model was also performed for comparison. Prespecified subgroup analysis was conducted according to gestational timing of NLR measurement, comparing studies measuring NLR in the first trimester or early pregnancy with studies measuring NLR at other time points. Leave-one-out sensitivity analysis, exclusion of the most influential study, exclusion of Gupta et al. (28), Baujat plot, and influence diagnostics were used to evaluate robustness and identify influential studies. Publication bias was assessed by visual inspection of funnel plots and by Egger’s and Begg’s tests (33, 34). Meta-regression was considered but was not used as a primary analysis because only 11 studies were included in the quantitative synthesis and key covariates were inconsistently reported across studies. Under these conditions, formal meta-regression would be statistically underpowered and potentially misleading. Analyses were performed using R and the metafor package (35).

## 3 Results

**Figure 1:**
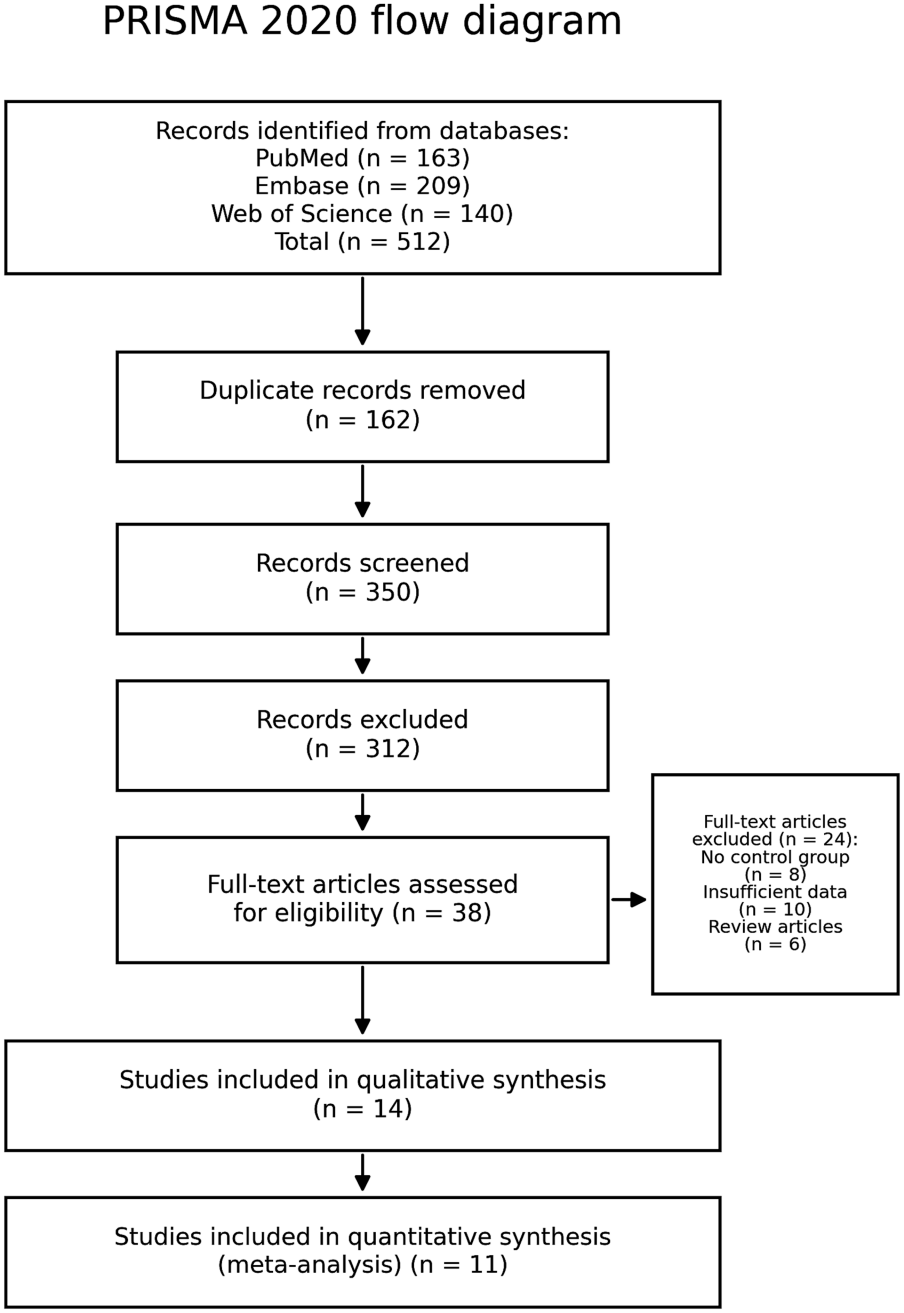
PRISMA flow diagram of study selection.

A total of 512 records were identified through database searching, including PubMed (*n* = 163), Embase (*n* = 209), and Web of Science (*n* = 140). After removing 162 duplicate records, 350 records remained for screening. Following title and abstract screening, 38 articles were assessed for full-text eligibility. Ultimately, 14 studies were included in the qualitative synthesis (15–28), of which 11 with extractable continuous NLR data were included in the quantitative meta-analysis, as shown in Figure 1. The main characteristics of the included studies are summarized in Table 1.

**Table 1:** Characteristics of the included studies (*n* = 14).

Study	Country	Design	Sample size (PE cases/controls)	Timing of NLR measurement
Kurtoglu et al., 2015 (15)	Turkey	Retrospective study	130/73	At diagnosis/late pregnancy
Kirbas et al., 2015 (16)	Turkey	Retrospective observational case–control study	614/320	First trimester (11–13 + 6 weeks)
Gezer et al., 2016 (17)	Turkey	Retrospective study	209/221	First trimester
Serin et al., 2016 (18)	Turkey	Case–control study	77/30	At diagnosis/late pregnancy
Jeon et al., 2017 (20)	South Korea	Retrospective study	68/86	At diagnosis/late pregnancy
Sachan et al., 2017 (22)	India	Prospective case–control study	50/51	13–20 weeks
Yucel and Ustun, 2017 (19)	Turkey	Retrospective cohort study	109/110	At diagnosis/late pregnancy
Kim et al., 2018 (21)	South Korea	Retrospective study	353/471	At diagnosis/late pregnancy
Gogoi et al., 2019 (23)	India	Cross-sectional study	67/67	At diagnosis/late pregnancy
Panwar et al., 2019 (24)	India	Prospective nested case–control study	64/376	16–18 weeks
Oglak et al., 2021 (25)	Turkey	Retrospective case–control study	201/100	First trimester (6–14 weeks)
Thombare et al., 2023 (26)	India	Case–control study	70/70	At diagnosis/late pregnancy
Sanjeewa 2024 (27)	Sri Lanka	Descriptive cross-sectional study	24/356	First trimester
Gupta et al., 2025 (28)	India	Prospective case–control study	30/70	Early pregnancy (12–16 weeks)

PE, preeclampsia; NLR, neutrophil-to-lymphocyte ratio.

Domain-level NOS scoring is provided in Supplementary Table S4. Available information on preeclampsia definition, severity classification, early- versus late-onset disease reporting, sampling timing, adjusted confounders, and key exclusion criteria is summarized in Supplementary Table S5. Raw extracted data for the quantitative synthesis are provided in Supplementary Table S6.

The included studies were mainly observational case–control studies and varied in sample size and timing of NLR measurement. According to the Newcastle-Ottawa Scale, most studies were of moderate to high methodological quality.

**Figure 2:**
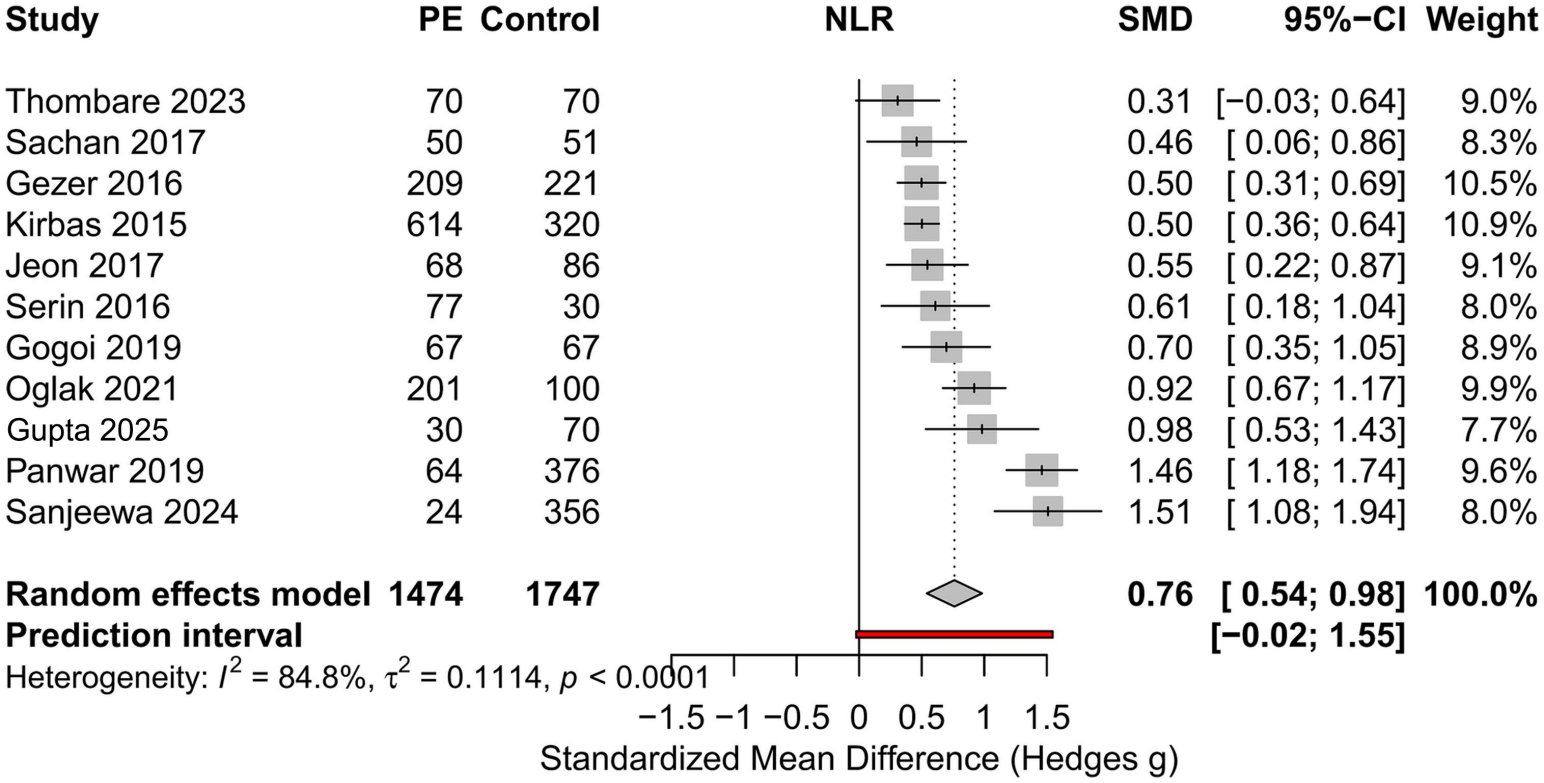
Forest plot of the association between neutrophil-to-lymphocyte ratio (NLR) and preeclampsia. Squares represent individual study estimates, horizontal lines indicate 95% confidence intervals, and the diamond represents the pooled random-effects estimate.

The random-effects meta-analysis of 11 studies demonstrated that maternal NLR levels were significantly higher in women with preeclampsia than in normotensive pregnant controls (SMD = 0.76, 95% CI 0.54–0.98; *I*^2^ = 84.8%; τ^2^ = 0.111), as shown in Figure 2 and Table 2. Results remained consistent under Hartung-Knapp adjustment (SMD = 0.76, 95% CI 0.49–1.03), REML estimation (SMD = 0.76, 95% CI 0.53–1.00; τ^2^ = 0.130), and Paule-Mandel estimation (SMD = 0.76, 95% CI 0.53–1.00; τ^2^ = 0.132). The fixed-effect model yielded a smaller but still significant estimate (SMD = 0.67, 95% CI 0.59–0.75). Exclusion of Panwar et al. (24), identified as the most influential study, reduced heterogeneity (*I*^2^ = 73.0%) but did not materially change the pooled effect size (SMD = 0.68, 95% CI 0.50–0.85). Exclusion of Gupta et al. (28), performed as an additional sensitivity analysis due to potential methodological and indexing-related concerns, also did not materially change the estimate (SMD = 0.74, 95% CI 0.51–0.98; *I*^2^ = 85.9%).

**Table 2:** Summary of pooled meta-analysis results.

Outcome	SMD (95% CI)	*p* value	*I*^2^ (%)	τ^2^
Overall analysis	0.76 (0.54–0.98)	<0.001	84.8	0.111
Hartung-knapp adjustment	0.76 (0.49–1.03)	<0.001	84.8	0.111
Excluding panwar 2019	0.68 (0.50–0.85)	<0.001	73.0	0.054
Fixed-effect model	0.67 (0.59–0.75)	<0.001	–	–

Eleven studies were included in the quantitative meta-analysis because sufficient continuous NLR data were available for pooling. Four studies measured NLR during the first trimester or early pregnancy and were therefore included in the first-trimester or early-pregnancy subgroup, whereas seven studies measured NLR at diagnosis or during later pregnancy.

**Figure 3:**
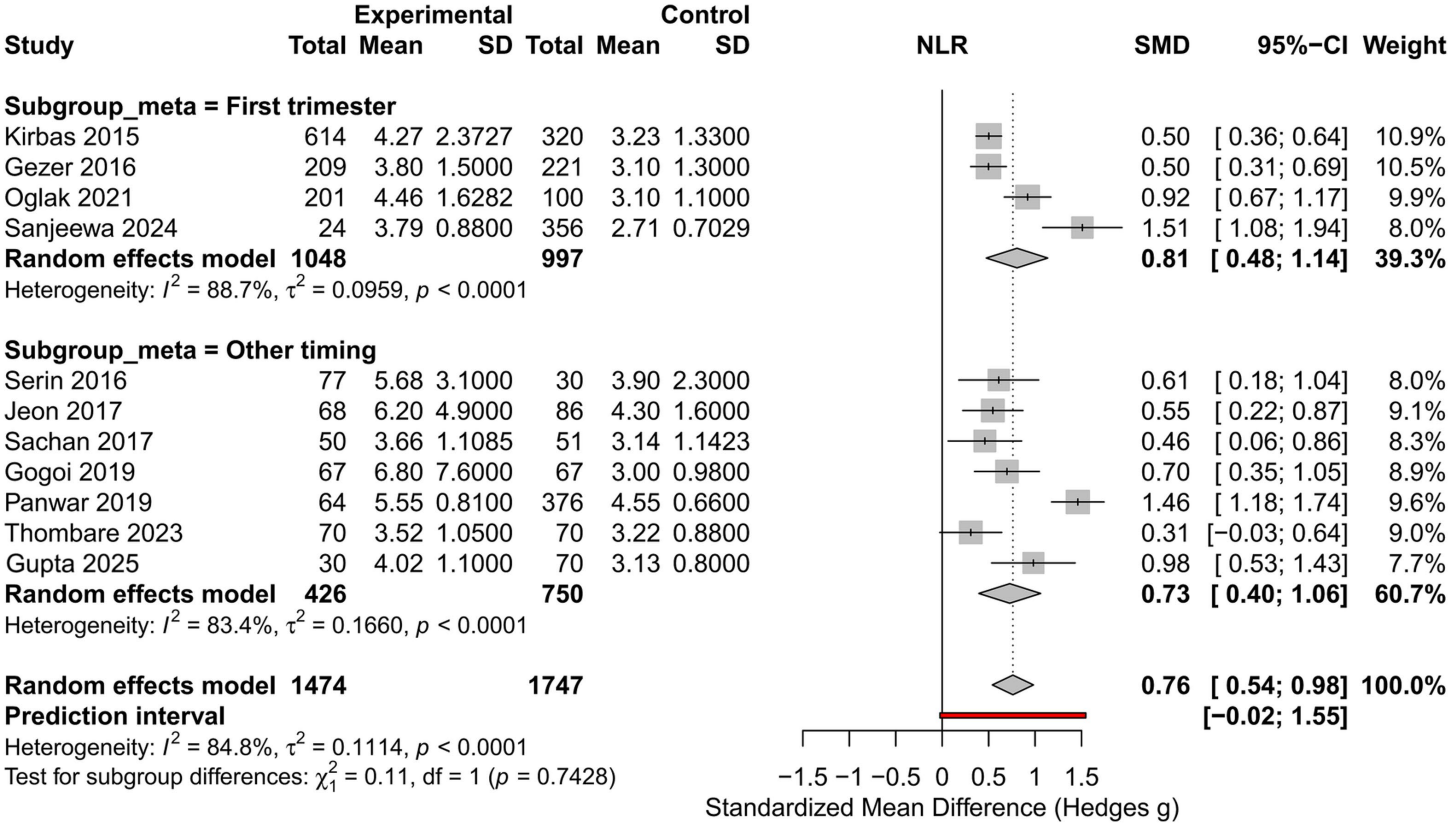
Forest plot of subgroup analysis according to gestational timing of NLR measurement.

**Figure 4:**
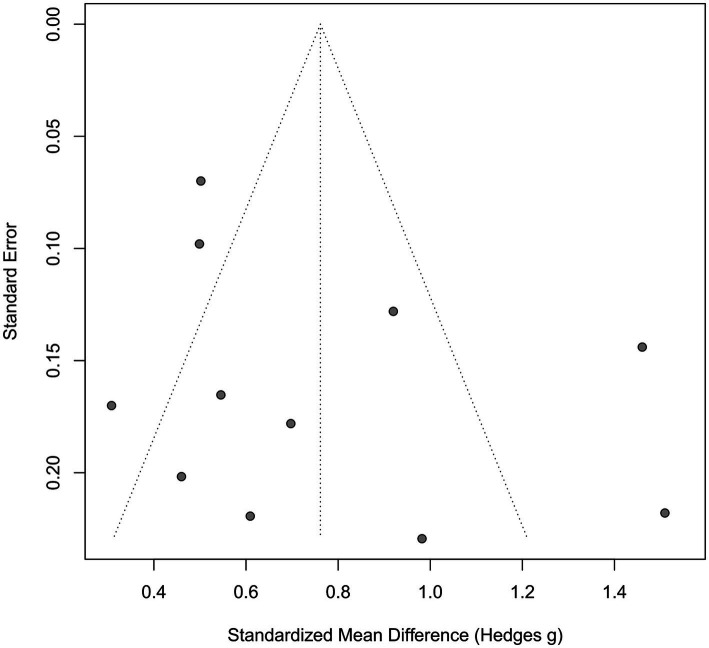
Funnel plot assessing potential publication bias for studies evaluating the association between neutrophil-to-lymphocyte ratio (NLR) and preeclampsia.

Subgroup analysis according to gestational timing indicated that the association remained significant in the first-trimester or early-pregnancy subgroup [4 studies: Kirbas et al. (16), Gezer et al. (17), Oglak et al. (25), and Sanjeewa (27); SMD = 0.81, 95% CI 0.48–1.14; *I*^2^ = 88.7%] and in studies measuring NLR at other time points (7 studies; SMD = 0.73, 95% CI 0.40–1.06; *I*^2^ = 83.4%), as shown in Figures 3, 4. The test for subgroup differences was not statistically significant (*p* = 0.743), although this comparison was limited by the small number of studies in each subgroup and should be interpreted cautiously.

Formal tests did not detect statistically significant publication bias (Egger’s *p* = 0.220; Begg’s *p* = 0.243). However, because only 11 studies were included, these tests had limited statistical power. Funnel plot inspection (Figure 4) suggested some asymmetry and possible small-study effects; therefore, publication bias cannot be excluded.

Leave-one-out sensitivity analysis demonstrated that the pooled effect size remained stable after sequential exclusion of individual studies (Supplementary Figure S1; Supplementary Table S1). Influence diagnostics and heterogeneity contributions were further explored using a Baujat plot and influence plot (Supplementary Figures S2, S3). Panwar et al. (24) contributed more prominently to heterogeneity; however, exclusion of this study did not materially change the pooled effect size, suggesting that the overall association was not driven by a single study. The finding should nevertheless be interpreted in light of substantial residual heterogeneity.

## 4 Discussion

This meta-analysis provides quantitative evidence that maternal neutrophil-to-lymphocyte ratio (NLR) levels are significantly elevated in women with preeclampsia compared with normotensive pregnancies. The overall pooled effect was moderate-to-large (SMD = 0.76); however, the substantial heterogeneity limits the precision and generalizability of the pooled estimate. These findings support an association between systemic inflammatory activation and preeclampsia (6–11), but they do not establish diagnostic accuracy or independent predictive performance.

The substantial heterogeneity observed in this meta-analysis should be considered a major limitation rather than a minor statistical issue. An *I*^2^ value of 84.8% indicates considerable between-study heterogeneity, and the τ^2^ value indicates meaningful between-study variability in standardized effect estimates. This variability is clinically plausible because the included studies differed in gestational age at blood sampling, timing of sampling relative to disease onset, diagnostic criteria, preeclampsia severity, early- versus late-onset disease reporting, study design, ethnicity, sample size, laboratory procedures, and adjustment for confounding. Accordingly, the pooled SMD should be interpreted as an overall association rather than a precise universal estimate applicable to all pregnant populations.

Biologically, the association between elevated NLR and preeclampsia is plausible because preeclampsia involves immune dysregulation, endothelial dysfunction, and exaggerated systemic inflammation. An increased NLR may reflect enhanced neutrophil-mediated innate immune activation with relative lymphocyte-mediated immune imbalance. However, NLR is an indirect and nonspecific marker and cannot establish specific mechanistic pathways.

NLR is also influenced by many non-preeclampsia-related conditions. Maternal obesity, subclinical infection, smoking, chronic hypertension, diabetes mellitus, autoimmune disease, corticosteroid exposure, labor status, aspirin prophylaxis, and gestational age at blood sampling may all affect NLR values. These variables were not consistently reported or adjusted for across the included studies, so residual confounding remains an important limitation.

The included studies addressed related but not identical clinical questions. Studies measuring NLR in early pregnancy are more relevant to potential risk prediction, whereas studies measuring NLR at diagnosis or in late pregnancy mainly reflect inflammatory status at or near disease onset. Therefore, the pooled estimate should be interpreted as evidence of an association between NLR and preeclampsia rather than proof of early predictive performance.

Our findings are broadly consistent with a previous meta-analysis evaluating the predictive role of NLR in preeclampsia (36). However, that analysis focused primarily on predictive performance, whereas the current study emphasizes the overall association between NLR levels and the presence of preeclampsia, incorporates recently published studies, and applies a more cautious classification of gestational timing of NLR measurement. Direct comparison with prior work should account for differences in study inclusion, timing of measurement, and whether the endpoint was association or prediction.

Subgroup analyses indicated that the association between NLR and preeclampsia was detectable both in studies measuring NLR in the first trimester or early pregnancy and in studies measuring NLR at later gestational time points. However, further subdivision into narrower gestational windows was not performed because the number of studies within each interval would have been too small for meaningful quantitative synthesis. The non-significant test for subgroup differences should therefore not be interpreted as evidence that gestational timing has no influence on NLR.

Although NLR can be obtained from routine complete blood counts and does not require specialized assays, the present meta-analysis did not synthesize diagnostic accuracy metrics, establish clinically actionable thresholds, compare NLR with established screening tools, or evaluate its incremental value in existing prediction models. Therefore, NLR should be considered a potentially useful adjunctive inflammatory marker for research rather than a stand-alone diagnostic or predictive tool for preeclampsia.

Several limitations should be considered. First, heterogeneity was substantial, and the pooled estimate should be interpreted as an overall association rather than a precise universal effect size. Second, the included studies differed in gestational age at sampling and clinical context; early-pregnancy studies are more relevant to prediction, whereas studies performed at diagnosis or in late pregnancy mainly reflect inflammatory status after or near disease onset. Third, diagnostic criteria, severity classification, and early- versus late-onset preeclampsia were not consistently reported. Fourth, residual confounding is likely because important factors such as BMI, infection, smoking, chronic hypertension, diabetes, autoimmune disease, corticosteroid exposure, labor status, aspirin prophylaxis, and gestational age at sampling were not uniformly adjusted for. Fifth, formal publication-bias tests had limited power because only 11 studies were included, and funnel plot asymmetry and small-study effects cannot be excluded. Sixth, this meta-analysis did not pool diagnostic accuracy metrics or establish clinically actionable NLR thresholds. Seventh, although no study had completed finalized, verified data extraction for quantitative synthesis before PROSPERO registration, preliminary literature screening and data organization had already been initiated. This may increase the risk of *post hoc* methodological decisions and should be considered when interpreting the findings. Therefore, the findings should not be interpreted as evidence that NLR can independently diagnose or predict preeclampsia.

## 5 Conclusion

Maternal NLR was significantly higher in women with preeclampsia than in normotensive pregnant controls, supporting an association between systemic inflammation and preeclampsia. However, substantial heterogeneity, differences in sampling timing, inconsistent reporting of disease severity, and limited adjustment for confounding restrict the clinical interpretation of this finding. NLR may provide adjunctive inflammatory information, but it should not be considered a stand-alone diagnostic or predictive marker. Future prospective studies should use standardized sampling windows, prespecified thresholds, consistent preeclampsia definitions, and diagnostic accuracy outcomes.

## Data Availability

All data generated or analyzed during this study are included in this article and its supplementary material. The raw extracted data used for quantitative synthesis are provided in Supplementary Table S6.

## Supplementary material

SUPPLEMENTARY FIGURE S1 Leave-one-out sensitivity analysis for the meta-analysis of the association between neutrophil-to-lymphocyte ratio (NLR) and preeclampsia.

SUPPLEMENTARY FIGURE S2 Baujat plot for identifying studies contributing to overall heterogeneity.

SUPPLEMENTARY FIGURE S3 Influence diagnostics for the meta-analysis of the association between neutrophil-to-lymphocyte ratio (NLR) and preeclampsia.
