# Supplementary figures and images for: Association between the neutrophil-to-lymphocyte ratio and preeclampsia: a systematic review and meta-analysis of observational studies
Source: Front Public Health. 2026 Jun 30;14:1865278. doi: 10.3389/fpubh.2026.1865278 (PMC13366881; doi:10.3389/fpubh.2026.1865278)

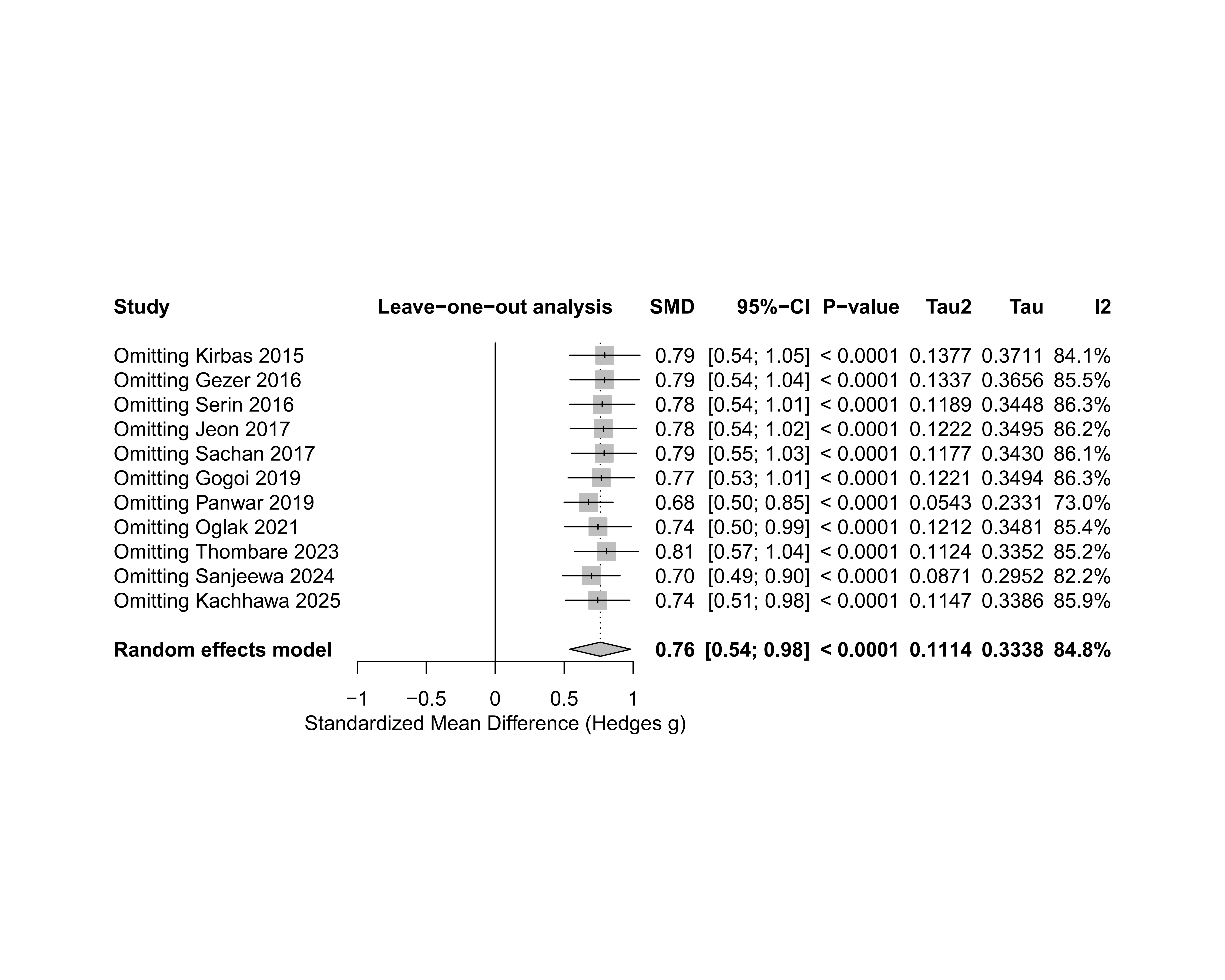

Supplement: SUPPLEMENTARY FIGURE S1 — Leave-one-out sensitivity analysis for the meta-analysis of the association between neutrophil-to-lymphocyte ratio (NLR) and preeclampsia. [file Image_1.PNG]

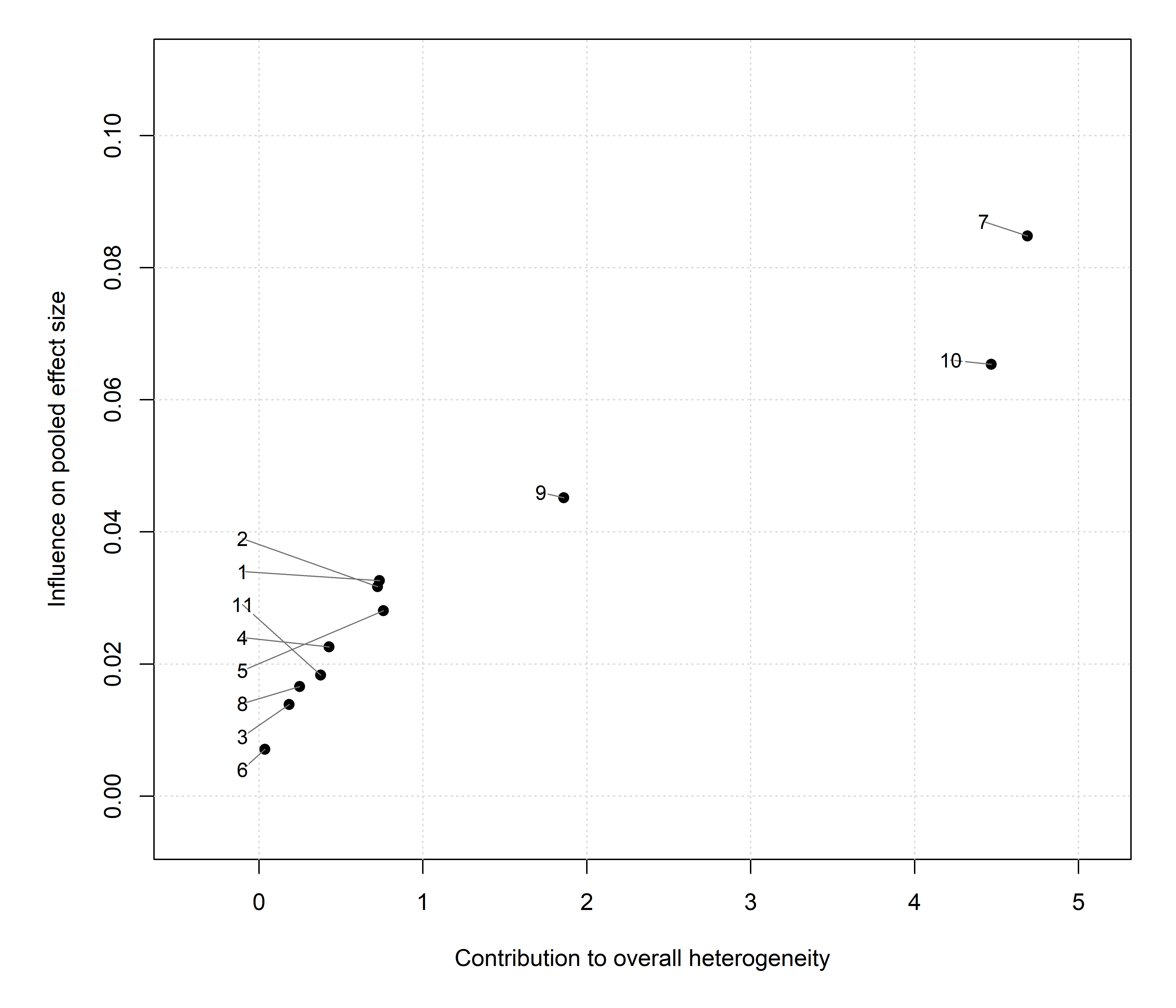

Supplement: SUPPLEMENTARY FIGURE S2 — Baujat plot for identifying studies contributing to overall heterogeneity. [file Image_2.TIFF]

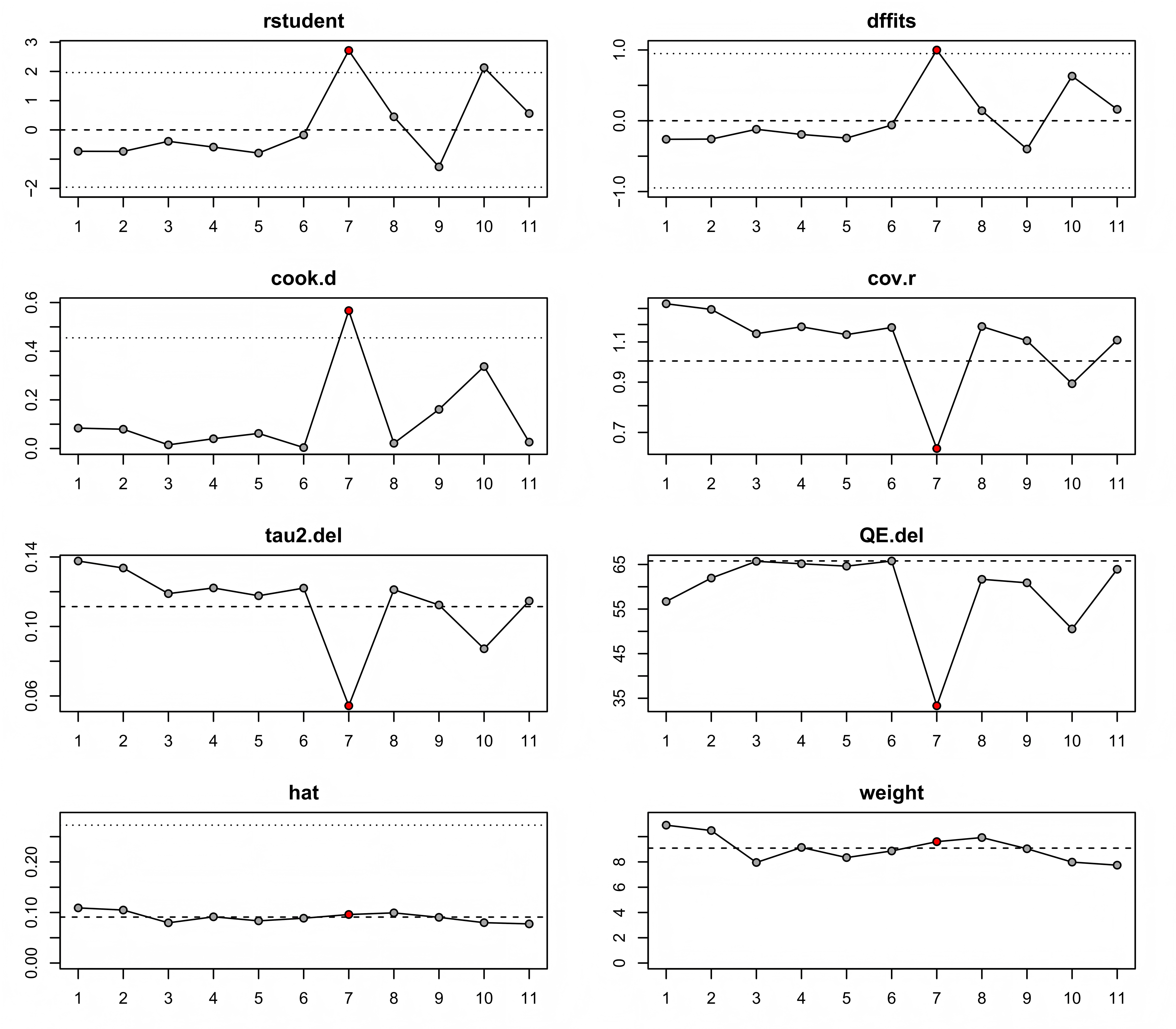

Supplement: SUPPLEMENTARY FIGURE S3 — Influence diagnostics for the meta-analysis of the association between neutrophil-to-lymphocyte ratio (NLR) and preeclampsia. [file Image_3.PNG]
